# Shift in hospital opioid use during the COVID-19 pandemic in Brazil: a time-series analysis of one million prescriptions

**DOI:** 10.1038/s41598-023-44533-5

**Published:** 2023-10-11

**Authors:** Romulo Mendonça Carvalho, Maria Clara de Magalhães-Barbosa, Lucas Monteiro Bianchi, Gustavo Rodrigues-Santos, Antônio José Ledo Alves da Cunha, Francisco Inácio Bastos, Arnaldo Prata-Barbosa

**Affiliations:** 1 Doctoral Program in Medical Sciences, D’Or Institute for Research & Education (IDOR), Rio de Janeiro, RJ 22281-100 Brazil; 2 Pharmaceutical Division, Rede D’Or São Luiz, Rio de Janeiro, RJ 22270-010 Brazil; 3 Department of Pediatrics, D’Or Institute for Research & Education (IDOR), Rio de Janeiro, RJ 22281-100 Brazil; 4 Doctoral Program in Epidemiology in Public Health, National School of Public Health Sergio Arouca (ENSP), Rio de Janeiro, RJ 21041-210 Brazil; 5 Doctoral Program in Collective Health, Institute of Social Medicine (IMS), State University of Rio de Janeiro (UERJ), Rio de Janeiro, RJ Brazil; 6 Department of Pediatrics, School of Medicine, Federal University of Rio de Janeiro (UFRJ), Rio de Janeiro, RJ 21044-020 Brazil; 7 Laboratory of Health Information, Institute of Scientific and Technological Communication and Information in Health, Oswaldo Cruz Foundation (IOC), Rio de Janeiro, RJ 21040-900 Brazil

**Keywords:** Health care, Public health, Epidemiology

## Abstract

The pronounced change in the profile of hospitalized patients during COVID-19 and the severe respiratory component of this disease, with a great need for mechanical ventilation, led to changes in the consumption pattern of some medicines and supplies. This time-series study analyzed the in-hospital consumption of opioids during the COVID-19 pandemic in 24 Brazilian hospitals compared to the pre-pandemic period. Data included 711,883 adult patients who had opioids prescribed. In 2020, the mean consumption was significantly higher compared to 2019 for parenteral fentanyl, enteral methadone, and parenteral methadone. It was significantly lower for parenteral morphine parenteral sufentanil, and parenteral tramadol. For remifentanil, it did not differ. The number of patients in 2020 was lower but the mean consumption was higher for fentanyl, parenteral methadone, and remifentanil. It was lower for enteral methadone and parenteral sufentanil. The consumption of parenteral morphine and parenteral tramadol was stable. There was a relevant increase in hospital consumption of some potent opioids during the COVID-19 pandemic in Brazil. These results reinforce the concern about epidemiological surveillance of opioid use after periods of increased hospital use since in-hospital consumption can be the gateway to the misuse or other than the prescribed use of opioids after discharge.

## Introduction

The COVID-19 pandemic has been one of the humanity’s most serious challenges in contemporary history and a major global public health emergency. As of February 2023, there were over six hundred million reported cases and almost seven million deaths worldwide. Brazil was one of the most heavily affected countries in the world, second only to the United States in deaths (almost 700,000) and fifth after the United States, India, France, and Germany in the number of reported cases (more than 36,9 million)^1^. The case-fatality rate (1.9%) is not among the lowest^1^, and overall in-hospital mortality ranging from 6 to 62%, depending on age group, has been reported^2,3^. However, the pandemic’s impact on nations, societies, and health system has obvious differences, as recently highlighted by a comprehensive cross-national analysis^4^.

The challenges posed by COVID-19 were both local and global. Pharmaceutical procedures and the portfolio of medicines have been dramatically affected by the pronounced change in the profile of hospitalized patients and the parallel shift in the characteristics of care delivered to them (both strongly correlated). The permanent demand for emergency and intensive care beds, postponement or cancellation of several routine procedures^5^, and elective interventions have seriously changed healthcare services, with major impacts on entire health systems. The global demand for personal protective equipment (PPE) and various medical supplies and devices, including those essential for intensive care, such as ventilators, sedatives, and analgesics, have placed a heavy strain on global and local supply chains^6^.

Opioids are key analgesic and sedative drugs used in adequately managing and caring for severe COVID-19 patients, most of whom are treated in intensive care units. Medicines such as fentanyl have been widely used as intravenous infusions to sedate patients, usually in combination with other medicines such as benzodiazepines (e.g., midazolam) or other therapeutic classes such as propofol and dexmedetomidine. Other opioids, like morphine and methadone, and administration routes other than intravenous (e.g., enteral, or subcutaneous) have also been used to reduce patient agitation and distress during mechanical ventilation^7^. However, the in-hospital use of opioids may just be a part of this quite complex system of opioid use, as it may be the introduction of the drug (and its effects) to patients and a way to a tendency to the continued and possible misuse of these substances after hospital discharge, facilitating medication diversion and eventually, malpractice by professionals who are not familiar with the subtle pharmacology and therapeutic decisions respecting opioids. By the way, they comprise most people working in internal medicine and related clinical and surgical specialties in most settings^8^. They may have contributed to the worrying epidemic of abusive use of opioids by the population, as observed in the last decade in the United States of America (USA)^9–11^. Although Brazil is not yet facing an epidemic like this, indirect data on the increase in opioid prescriptions, misuse, and abuse of opioids and other substances in the country are worrisome^12–15^.

During COVID-19, there was a national guideline on the use of opioids in hospitalized patients with severe forms of this disease, especially needing mechanical ventilation, recommending preferentially the use of potent opioids with short half-life, such as fentanyl and remifentanil, by continuous infusion, which could be replaced by morphine in case of shortage of the recommended drugs^16^.

This study aimed to analyze the in-hospital use of opioids in a large network of hospitals in Brazil in 2020, during the COVID-19 pandemic, comparing it with the previous year. In addition, we reflect on the possible public health consequences of the increased non-medical use of these drugs in a very complex scenario in which in-hospital use is only a part of the problem, recognizing that, unfortunately, a comprehensive follow-up of patients after hospital discharge is usually absent, as happens in most settings and health systems, precluding sound conclusions about this critical interface.

## Results

The data included 711,883 hospitalizations of patients for whom opioids were prescribed and used. Its distribution by hospitals and states is shown in Table S1 and by sex and age group in the Table S2 (Supplementary file online).

**Figure 1:**
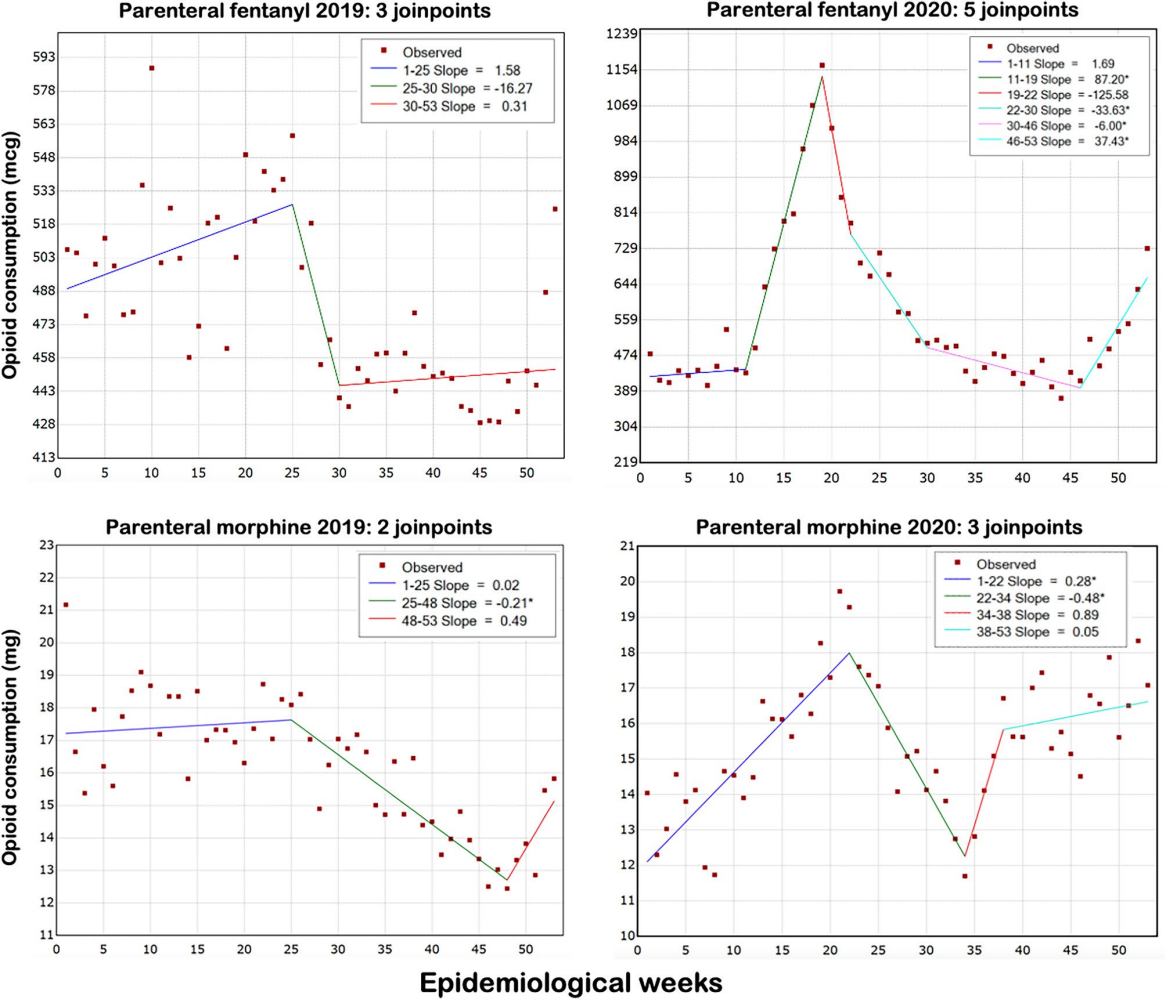
Time-series analysis of in-hospital parenteral fentanyl and parenteral morphine consumption in the 2019 and 2020 epidemiological weeks. The trend lines show the number of *joinpoints* selected by the model for each drug and year. Asterisks indicate that the respective slope significantly differs from zero at the alpha = 0.01 level.

**Figure 2:**
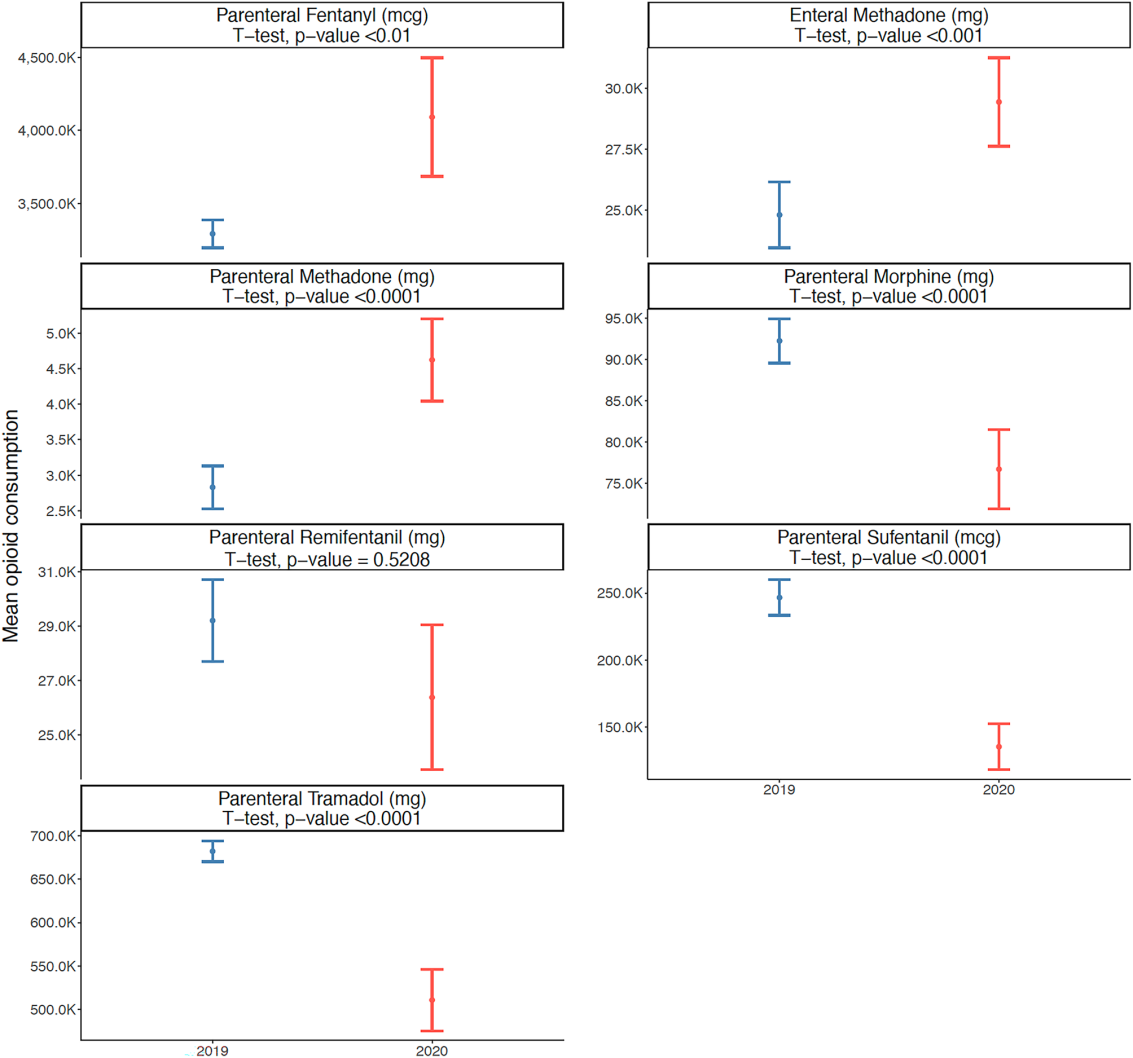
Mean in-hospital consumption of opioids in 2019 and 2020. The bars show the means and their 95% confidence interval. Data were compared using the Student t-test.

The use of parenteral fentanyl showed an increase in the total consumption in 2020 compared to 2019 (216,817,273 × 171,240,825 mcg). In 2019, consumption was stationary, with the highest mean weekly consumption being 588 mcg in EW 10. On the other hand, in 2020, there were two significant upward trends in total consumption, coinciding with the two waves of Covid-19 (gamma and delta variants of SARS-CoV-2). The first slope was between EW 11 and 19, with the highest mean weekly consumption reaching 1165 mcg in EW 19, and the second increase between EW 46 and 53, with the highest mean weekly consumption of 729 mcg in EW 53 (Table 1 and Fig. 1). The mean consumption of parenteral fentanyl by all hospitals was significantly higher in 2020 compared to 2019 (4,090,892 ± 1,508,169 vs. 3,293,093 ± 353,450 mcg; p < 0.01) (Table 1 and Fig. 2).

**Table 1:** Total opioid consumption in 2019 and 2020.

Drug and route	Units of consumption	Year	Total	Mean	Standard deviation	Highest mean weekly consumption
Parenteral fentanyl*	mcg	2019	171,240,825	3,293,093	353,450	588 (EW 10)
	mcg	2020	216,817,273	4,090,892	1,508,169	1165 (EW 19)
Enteral methadone*	mg	2019	1,289,378	24,796	5003	58 (EW 30)
	mg	2020	1,560,053	29,435	6756	60 (EW 40)
Parenteral methadone*	mg	2019	147,158	2830	1122	33 (EW 34)
	mg	2020	245,038	4623	2153	63 (EW 13)
Parenteral morphine^¶^	mg	2019	4,797,794	92,265	9962	21 (EW 1)
	mg	2020	4,066,550	76,727	17,782	20 (EW 21)
Parenteral remifentanil	mg	2019	1,518,910	29,210	5571	17 (EW 18)
	mg	2020	1,398,366	26,384	9896	29 (EW 19)
Parenteral sufentanil^¶^	mcg	2019	12,824,426	246,624	49,160	594 (EW 26)
	mcg	2020	7,183,737	135,542	63,912	495 (EW 24)
Parenteral tramadol^¶^	mg	2019	35,480,617	682,320	44,522	58 (EW 1)
	mg	2020	27,068,579	510,728	131,969	59 (EW 53)

*Significant increase in mean consumption between 2019 and 2020 (Student t test).

^¶^Significant decrease in mean consumption between 2019 and 2020 (Student t test).

**Figure 3:**
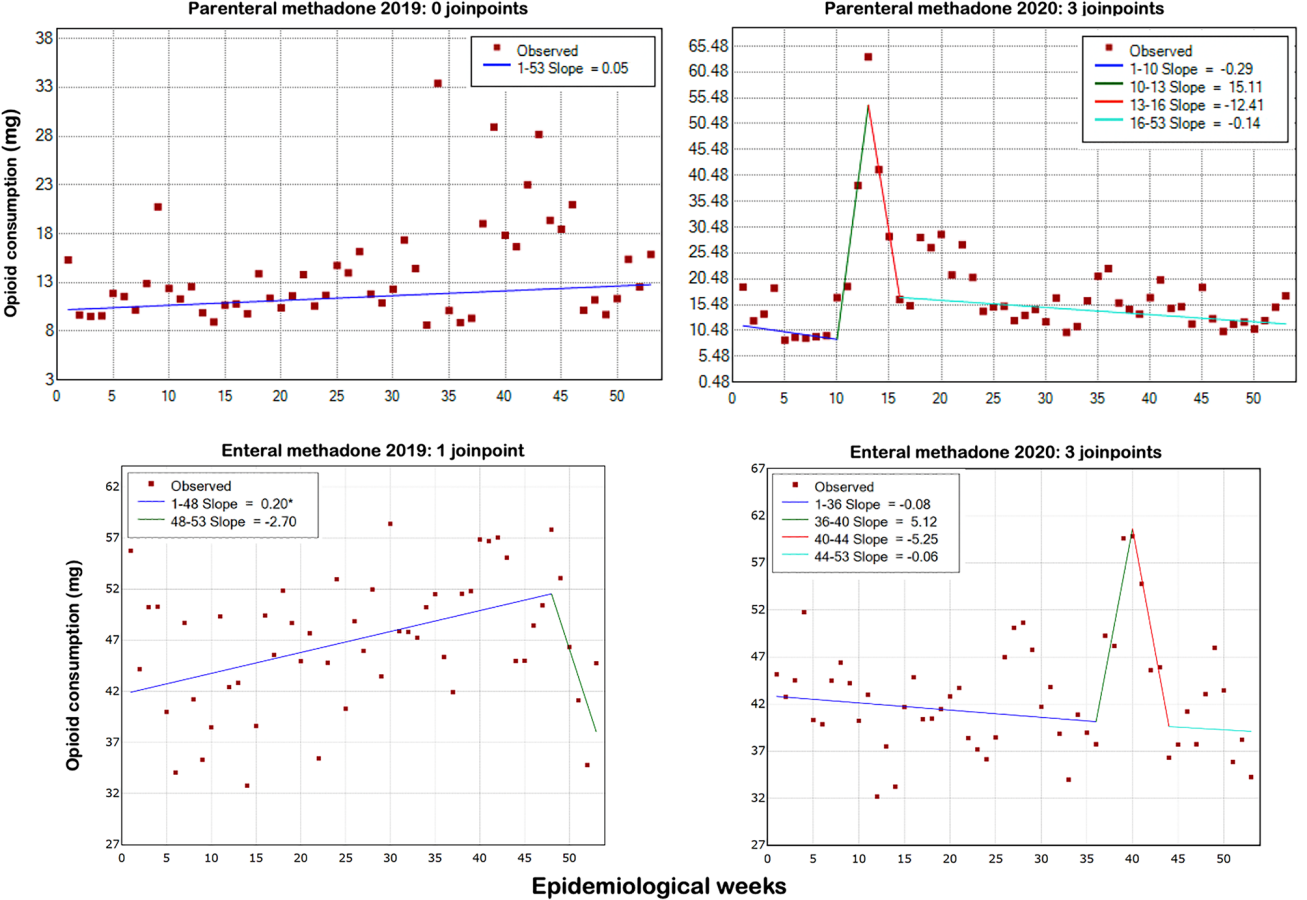
Time-series analysis of in-hospital parenteral and enteral methadone consumption in the 2019 and 2020 epidemiological weeks. The trend lines show the number of *joinpoints* selected by the model for each drug and year. Asterisks indicate that the respective slope significantly differs from zero at the alpha = 0.01 level.

A similar pattern was observed for parenteral morphine and parenteral methadone, which showed upward trends in 2020, coinciding with the first wave of Covid-19, between EWs 1–22 (morphine, Fig. 1, significant trend) and EWs 10 and 13 (methadone, Fig. 3, not significant trend). Especially for parenteral methadone, the highest mean weekly dose in 2020 (63 mg in EW 13) was much higher than in 2019 (33 mg in EW 34). However, the total consumption of parenteral morphine was lower in 2020 (4,066,550 × 4,797,794 mg), as well as the mean consumption (76,727 ± 17,782 × 92,265 ± 9,962 mg; p < 0.001, Table 1 and Fig. 2). For parenteral methadone, the total and mean consumption were higher in 2020: 245,038 × 147,158 mg, and   4623 ± 2153 × 2830 ± 1122 mg; p < 0.0001, Table 1 and Fig. 3). There was a peak in enteral methadone consumption between EW 35–40 in 2020, coinciding with the significant decrease in parenteral morphine use, but this trend did not reach statistical significance. There was also an increase in total consumption of enteral methadone in 2020 (1,560,053 × 1,289,378 mg), with a significantly higher mean consumption (29,435 ± 6,756 × 24,796 ± 5003 mg; p < 0.001) (Table 1 and Fig. 3). The trend analysis showed a significant upward patter in 2019 but not in 2020 (Fig. 3).

**Figure 4:**
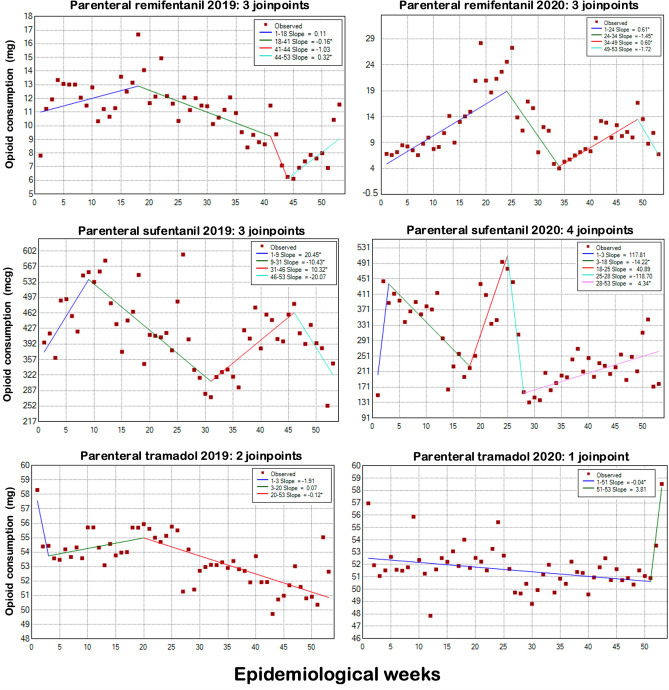
Time-series analysis of in-hospital parenteral consumption of remifentanil, sufentanil, and tramadol in the 2019 and 2020 epidemiological weeks. The trend lines show the number of *joinpoints* selected by the model for each drug and year. Asterisks indicate that the respective slope significantly differs from zero at the alpha = 0.05 level.

The remifentanil consumption was lower in 2020 (mean: 26,384 ± 9896 vs. 29,210 ± 5,571 mg; total: 1,398,366 × 1,518,910 mg) as showed in Table 1 and Fig. 2, but these differences did not reach statistical significance. The trend analysis showed a significant downward trend in both years, but especially between EWs 24 and 34 in 2020, coinciding with the effects of the first wave of Covid-19 (Fig. 4). The use of parenteral sufentanil showed an oscillatory pattern in both years (Fig. 4), but with a much higher total (12,824,426 × 7,183,737 mcg) and mean consumption in 2019 (246,624 × 135,542 mcg; p < 0.0001) (Table 1 and Fig. 2). The use of parenteral tramadol showed a decrease from 2019 to 2020 (35,480,617 × 27,068,579 mg), reflected by the significantly higher mean consumption in 2019 compared with 2020 (682,320 ± 44,522 × 510,728 ± 131,969; p < 0.001) (Table 1 and Fig. 2). A significant downward trend was observed in both years (Fig. 4).

**Figure 5:**
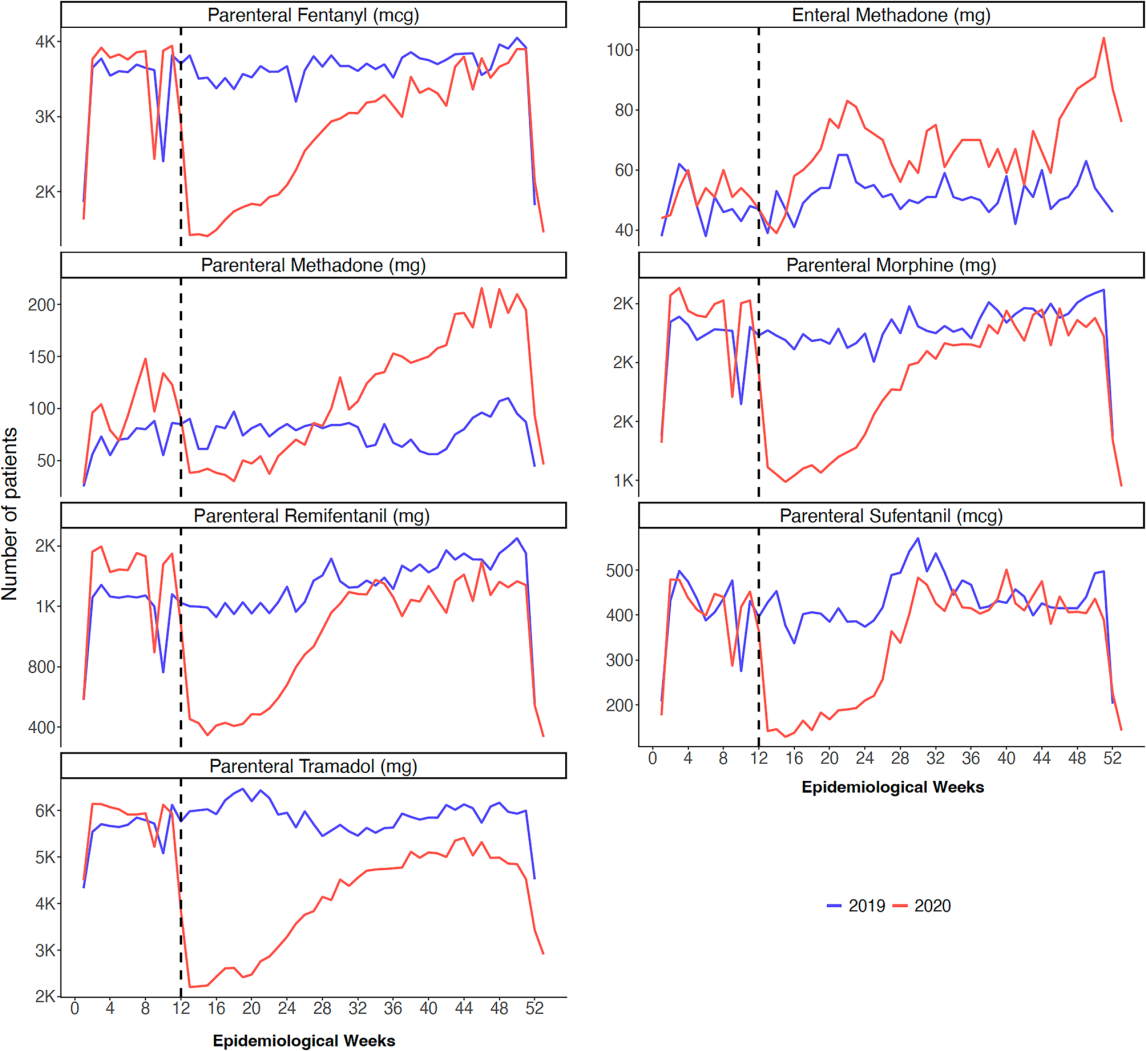
Time-series analysis of the number of hospitalized patients that received opioids, according to active ingredients and administration routes, over the epidemiological weeks of 2019 and 2020. The horizontal axis shows epidemiological weeks, and the vertical axis indicates the number of patients according to active ingredients and administration routes. The dotted line represents epidemiological week 12 and marks Brazil's first confirmed COVID-19 case.

The absolute number of patients included in 2020 was lower than in 2019 (310,281 × 401,602) (Table 2 and Fig. 5). Despite this reduction, mean opioid consumption per patient compared to 2019 was higher for fentanyl (1410 × 920 mcg/patient), parenteral methadone (42 × 37 mg/patient), and remifentanil (25 × 22). On the other hand, it was lower for enteral methadone (452 × 487 mg/patient) and parenteral sufentanil (395 × 579 mg). For parenteral morphine (40 × 41 mg/patient) and parenteral tramadol (117 × 117 mg/patient), consumption was equivalent (Table 2).

**Table 2:** Number of patients consuming opioids per type of opioid in the years 2019 and 2020.

Drug and route	Units of consumption (UC)	Year^a^	Patients, n (%)	Total consumption in the year (UC)	Mean consumption per patient in the year (UC/patient)
Parenteral fentanyl	mcg	2019	186,159 (46.4)	171,240,825	920
	mcg	2020	153,795 (49.6)	216,817,273	1410
Enteral methadone	mg	2019	2650 (0.7)	1,289,378	487
	mg	2020	3449 (1.1)	1,560,053	452
Parenteral methadone	mg	2019	3962 (1.0)	147,158	37
	mg	2020	5810 (1.9)	245,038	42
Parenteral morphine	mg	2019	117,767 (29.3)	4,797,794	41
	mg	2020	101,463 (32.7)	4,066,550	40
Parenteral remifentanil	mg	2019	67,753 (16.9)	1,518,910	22
	mg	2020	56,083 (18.1)	1,398,366	25
Parenteral sufentanil	mcg	2019	22,135 (5.5)	12,824,426	579
	mcg	2020	18,177 (5.9)	7,183,737	395
Parenteral tramadol	mg	2019	302,025 (75.2)	35,480,617	117
	mg	2020	232,341 (74.9)	27,068,579	117

^a^Total number of patients: 2019 = 401,602; 2020 = 310,281.

*UC* units of consumption.

## Discussion

The study found that the in-hospital consumption of some potent intravenous opioids, especially fentanyl and methadone, increased during the COVID-19 pandemic at participating hospitals. In parallel, there was also a significant rise in the consumption of enteral methadone, a drug generally used to discontinue the prolonged use of parenteral opioids. These findings coincided with the increase in hospitalizations due to COVID-19 in Brazil in two waves, at the beginning and end of 2020. On the other side, the in-hospital consumption of morphine, sufentanil, and tramadol, all administered parenterally, showed a significant reduction in 2020 compared to the same period in 2019.

The ventilatory assistance in patients with severe acute respiratory syndrome due to COVID-19, especially those requiring orotracheal intubation, often followed by tracheostomy, may explain the increased consumption of potent intravenous opioid analgesics, such as fentanyl and methadone, and, consequently, also oral methadone, the natural discontinuation drug. On the other side, the drastic reduction (approximately 50%) in elective and low-complexity surgical procedures during the COVID-19 pandemic^5^ may explain the abrupt drop in the consumption of opioids like morphine and tramadol, widely used for postoperative analgesia, as well as sufentanil, widely used as an adjuvant analgesic in spinal anesthesia procedures. The increase in in-hospital consumption of fentanyl and methadone in 2020, despite the overall decrease in hospitalized patients in the same period, compared to 2019, corroborates the use of higher doses of these drugs.

It is essential to distinguish between the prevailing scenarios in countries such as Brazil and the United States. Brazil has not experienced a large-scale opioid crisis^12^ like the one affecting the United States in recent years^17^. In Brazil’s drug scene, powder cocaine and other coca paste derivates (e.g., crack cocaine and related smoked presentations) are highly prevalent. Until recently, the challenge posed by the non-therapeutic use of opioids was basically ignored^12–14^. Recent increases in non-therapeutic opioid use in Brazil pale in comparison to the opioid epidemic observed in countries like the United States^15,17–19^. For this reason, and due to the virtual absence of heroin and other opioids like fentanyl on the Brazilian drug scenes for decades, Brazil does not have dedicated programs for the management and care of persons with opioid use disorder, such as methadone substitution therapy. However, a recent study demonstrated that 37% of older adults in Brazil reported chronic pain, and 30% use opioids, higher among females and single individuals, calling attention to the need for a surveillance program of opioid prescription to prevent harmful consequences^20^.

However, there is no room for complacency. The in-hospital use of potent opioids in the context of an extensive and prolonged period such as the COVID-19 pandemic calls for maximum attention. Unfortunately, at the beginning of the pandemic, there was a lack of understanding of the disease, and many patients were intubated due to fear of using non-invasive ventilation in this context, resulting in more extended periods of sedation than usual. The unexpected increase in the use of a group of potent opioids during the hospitalization of COVID-19 patients poses a major challenge in terms of adverse effects secondary to their therapeutic use^21^, as well as proper management after hospital discharge. Two key factors are relevant here. First, this group of patients has used substantial amounts of opioids in the context of emergency and critical care, where the health staff is focused on the pressing need to save lives during a fast-spreading epidemic. In addition, delirium is a possible neurological manifestation of SARS-Cov2 infection, or a consequence of prolonged ICU stay under adverse conditions of isolation imposed by COVID-19, associated with the use of sedatives. Furthermore, this entire context predisposes to the onset of post-traumatic stress disorder after hospital discharge. The use of neuroleptic or antidepressant drugs to treat these events may be necessary^22,23^. Patient management after hospital discharge has been a relevant need, still to be properly addressed. Assessments of real-world facilitators to prescribing buprenorphine/naloxone (BUP) in the emergency department, as recently conducted by Dong et al.^24^, do not exist in Brazil. It is not clear whether patients with previous opioid-related problems or opioid-naive patients who have been medicated with potent opioids, sedatives, neuroleptic, and antidepressant drugs have been properly managed vis-à-vis the development of untoward consequences, including opioid diversion, the progressive development of opioid use disorder, and the concomitant use of different medicines and substances that characterize polysubstance use disorder. The latter was found to be rare before the COVID-19 epidemic. Unfortunately, no updated information is available from population-based studies, but recent analyses highlight that polysubstance use disorder was an infrequent but non-negligible issue in Brazil before the COVID-19 epidemic^14^. A second dimension of the current challenge is the proper management of post-COVID syndrome, which tends to affect different organs and physical and mental functions and may be associated with pain. International protocols have been launched^25^, but to the best of our knowledge, nothing has been implemented in Brazil, and the management of chronic pain of patients affected by post-COVID syndrome has been tentative. Opioids should be used with maximum caution to avoid the combined use and potential misuse of opioids during and after hospitalization.

This study has some strengths and limitations. We were able to standardize the prescriptions of a wide range of different opioids with various presentations and develop a systematic analysis of the in-hospital consumption of these drugs. The magnitude of the data and the standardization of all opioid drugs under study are of great relevance for the care of hospitalized patients and make important contributions to understanding the impact of the COVID-19 pandemic. However, there were some limitations related to information bias. First, the use of secondary data may have been associated with inherent information bias in this study type. However, data was carefully checked for consistency, and eventual findings were reviewed and corrected. Second, the data collected did not include clinical variables, such as indications for use, clinical conditions that determined the consumption pattern, such as the need to change doses, signs of dependence or withdrawal, etc., preventing the exploratory analysis of associations of opioids consumption with the patient's clinical picture. Third, the study was conducted only in private hospitals, which could raise the question of its generalizability. However, regardless of the type of hospital—private or public—during the pandemic, care protocols were standardized by medical societies and the Ministry of Health, which increases the probability of generalizing the results found.

In conclusion, this study documents the 2020 pandemic’s significant impact on the profile of in-hospital opioid consumption. Although it cannot infer an increase in out-of-hospital opioid use, as participants were not followed up after discharge, it reinforces the concern about epidemiological surveillance of opioid use after periods of increased hospital use. Although Brazil is not dealing with an epidemic situation of opioid misuse^13^ like that in the United States, the impact of opioids used for medical purposes on overall opioid consumption is always a central part of the issue^8,26^. Moreover, the possible future repercussions of these findings are of concern. Although Brazil is not currently experiencing a problem with heroin as a street drug, the substantial change in prescription patterns and the relevant increase in in-hospital consumption of potent opioids pose a challenge for the country’s immediate future, not only during the COVID-19 pandemic but also in the coming period, with the potential for increasing numbers of individuals with substance use disorder.

## Methods

All methods were performed in accordance with the relevant guidelines and regulations, especially the REporting of studies Conducted using Observational Routinely-collected Data (RECORD) Statement^27^.

### Study design, population, and setting

The study consists of a time-series analysis. Data on opioid prescriptions collected during the COVID-19 epidemic (2020) were cross-compared with opioid prescription patterns before the pandemic’s emergence in Brazil (2019). All hospitalized patients 18 years or older who had opioids prescribed in the study period were included. The data are from 24 hospitals in Brazil: 17 in the Southeast region (Rio de Janeiro, 9 and São Paulo, 8), four in the Northeast (Pernambuco), and three in the Center region (Federal District), which are part of the largest network of private hospitals in Brazil (Rede D’Or São Luiz). In the study period, it consisted of 69 large referral hospitals distributed across nine states and the Federal District (Brasília). It has 11,000 beds, more than 800,000 hospitalizations, and 360,000 surgeries annually^28^. Data were distributed by Epidemiological Weeks (EW): the 52/53 weeks of each year (2019 and 2020). This allowed for the standardization of the distribution of variables in weekly units, respecting the international epidemiological calendar.

### Study variables

The study variables were hospitalization, patient medical record code, year of admission, hospital sector, opioid prescription, opioid consumption, and epidemiological week (EW). We studied the prescription, dispensing, and use of selected opioids: parenteral fentanyl, parenteral methadone, enteral methadone, parenteral morphine, parenteral remifentanil, parenteral sufentanil, and parenteral tramadol. The selection of a given subset of opioids was based on their inclusion in the Group of Opioid Analgesics, as defined by the Anatomical Therapeutic Chemical (ATC) list of the World Health Organization^29^, as well as on their relevance for management and care of hospitalized patients. Therefore, we did not include typical outpatient treatment formulations, such as oral presentations of morphine, tramadol, oxycodone, and transdermal patch presentations. Different brands of opioids with the same active ingredient were grouped and stratified according to their administration route (e.g., oral, intravenous, etc.). A defined daily dose (DDD), as defined by the World Health Organization (WHO), was standardized for each group of opioids, considering both the pharmaceutical formulation of the various active ingredients and the presentation in milligrams or micrograms.

### Data sources

All data on opioid prescriptions were extracted from the electronic management systems of the 24 participating hospitals. The institutions use two different e-management systems: Philips Tasy EMR™,Amsterdam, The Netherlands (https://www.philips.ae/healthcare/resources/landing/tasy) and WPD™ DGS Brasil—Dedalus Group, Barueri, Brazil (https://www.dedalusgroup.com/brasil/pt-pt) (Table S3, Supplementary file online).

Deterministic algorithms extracted data without additional interference by the researchers. All patient data, including drug prescriptions, had been primarily recorded in the individual medical records before being entered into the e-management systems. Data on daily opioid prescriptions were standardized, integrated, and transcribed into a previously defined spreadsheet. Algorithm procedures and data auditing by the study team were used for consistency checking.

This is a census study, summarizing the entire dataset of these 24 hospitals. No additional sampling procedure has been performed other than the purposeful selection of the initial units. The option for a convenience sample of a nonrandom subset of 24 hospitals distributed across three states and the Federal District rather than a probability sample was not fortuitous but deliberated, focusing on long-established hospitals, considering the fast-growing network with different levels of coverage and consistency of its electronic management systems. In the inevitable trade-off between consistency and comprehensiveness of management systems per unit versus generalizability, our choice privileged the former.

### Statistical methods

For the time series analysis, we used the software *Joinpoint Regression Program*, version 4.9.0 (Statistical Research and Applications Branch, Surveillance Research Program, National Cancer Institute, USA), which uses the technique of regression by inflection points (“joinpoints”), a segmented linear regression with correction for first-order autocorrelation, in which changes in the correlation between opioid consumption (dependent variable) and epidemiological weeks (independent variable) are connected by joinpoints^30^. The Weekly Percent Change (WPC) and their respective 99% confidence intervals were calculated for the generated *joinpoints*. WPC is one way to characterize trends in opioid use rates over time. With this approach, opioid use rates are assumed to change at a constant percentage of the rate of the previous week. One advantage of characterizing trends this way is that it is a measure that is comparable across scales, for different classes of opioids and units of measure. The various segments generated are explored through Monte Carlo permutation tests to choose the model that best explains the trend over time, starting from a minimum number of inflection points (e.g., 0 *joinpoints*, which is a straight line) and testing whether more joinpoints are statistically significant and must be added to the model. This enables to test that an apparent change in trend is statistically significant. The model selection was performed by the permutation test. This test is used recurrently for comparing two different *joinpoint* models, a simpler model with fewer *joinpoints* versus a more complex one, called alternative model. To test if the more complex model is better than the original, we calculate a ratio SSE_N_ /SSE_A_, where SSE_N_ is the sum of squared errors (SSE) from the null model and SSE_A_ is the equivalent of the alternative model. If the true model were the alternative model, we would expect that after permuting the errors most of the new ratios were less than the original ones. The program uses the grid search of Lerman (1980) to fit the segmented regression function and the p-value of each permutation test is estimated using Monte Carlo methods, and the overall asymptotic significance level is maintained through a Bonferroni adjustment^31–33^. The statistical significance level for the permutations test was set at 0.01. Significant values represent an increasing trend if the value is positive and decreasing trend if negative. Non-significant values represent a stationary (stable) trend. Since the observed data correspond only to two years being used for comparison, seasonality was not verified because it does not apply to relatively short periods with no discernible seasonal pattern^34^. The Student t-test was used to test the hypothesis of a significant difference between the average consumption of each opioid in 2019 and 2020. The significance level was set at 0.01. All the other descriptive cross-tabulations and statistical analysis were performed with the R 4.1.0 (R Foundation for Statistical Computing, Vienna, Austria).

### Ethical aspects

The study protocol was approved by the Research Ethics Committee of the D’Or Institute for Research and Education under the number CAAE 23030719.7.0000.5249. As the study used retrospective data and the results would be presented in aggregated form, the same Ethics Committee waived the informed consent.

### Supplementary Information

Supplementary Information.

## Data Availability

Anonymized datasets may be requested from the corresponding author and are fully transparent and reproducible in full compliance with the standards and procedures summarized by Iqbal et al. (2006) and several other publications and statements^35^.
