# Supplementary material for: Shift in hospital opioid use during the COVID-19 pandemic in Brazil: a time-series analysis of one million prescriptions
Source: Sci Rep. 2023 Oct 11;13:17197. doi: 10.1038/s41598-023-44533-5 (PMC10567754; doi:10.1038/s41598-023-44533-5)
Supplement: Supplementary file 1 — Supplementary Information. [file 41598_2023_44533_MOESM1_ESM.pdf]

Supplementary file

Shift in hospital opioid use during the COVID-19 pandemic in Brazil: a time-series analysis of one million prescriptions

S1Table. Distributionofpatientsaccordingtostatesandparticipatinghospitalsin2019and2020.

	**2019**<br>**n (%)**	**2020**<br>**n (%)**
**Pernambuco**	**57932 (14.43)**	**39592 (12.76)**
São Marcos Hospital	10497 (2.61)	6864 (2.21)
Memorial São José Hospital	13714 (3.41)	9369 (3.02)
Esperança Recife Hospital	18242 (4.54)	12989 (4.19)
Esperança Olinda Hospital	15479 (3.85)	10370 (3.34)
**Distrito Federal**	**40060 (9.98)**	**31618 (10.19)**
Santa Helena Hospital	18815 (4.68)	15612 (5.03)
Santa Luzia Hospital	19175 (4.77)	14313 (4.61)
Coração do Brasil Hospital	2070 (0.52)	1693 (0.55)
**Rio de Janeiro**	**124501 (31.00)**	**95395 (30.74)**
Bangu Hospital	7892 (1.97)	6354 (2.05)
Barra D'Or Hospital	12899 (3.21)	9828 (3.17)
Caxias D'Or Hospital	19524 (4.86)	14635 (4.72)
Copa D'Or Hospital	15485 (3.86)	10118 (3.26)
Niterói D'Or Hospital	5327 (1.33)	6978 (2.25)
Norte D'Or Hospital	10322 (2.57)	8474 (2.73)
Oeste D'Or Hospital	21557 (5.37)	15254 (4.92)
Quinta D'Or Hospital	19550 (4.87)	13544 (4.37)
Rios D'Or Hospital	11945 (2.97)	10210 (3.29)
**São Paulo**	**179109 (44.60)**	**143676 (46.31)**
Assunção Hospital	12984 (3.23)	11117 (3.58)
Brasil Hospital	29651 (7.38)	21839 (7.04)
IFOR Hospital	10658 (2.65)	9151 (2.95)
Villa Lobos Hospital	14692 (3.66)	11766 (3.79)
Anália Franco Hospital	31081 (9.75)	24889 (8.02)
Itaim Hospital	39156 (9.75)	31486 (10.15)
Morumbi Hospital	25008 (6.23)	20227 (6.52)
São Caetano Hospital	15898 (3.95)	13201 (4.25)
**Total Patients**	**401602 (100.00)**	**310281 (100.00)**

Supplementary file

Shift in hospital opioid use during the COVID-19 pandemic in Brazil: a time-series analysis of one million prescriptions

S2 Table. Distribution of hospitalized patients by gender and age group.

Year	Gender	Age group (years)	Patients n	Patients %
2019	Female	18 - 39	120,528	29.97
		40 - 59	91,572	22.77
		60 - 79	32,575	8.10
		> 80	10,416	2.59
	Male	18 - 39	53,930	13.41
		40 - 59	61,008	15.17
		60 - 79	25,819	6.42
		> 80	6,314	1.57
2020	Female	18 - 39	100,969	32.60
		40 - 59	66,435	21.45
		60 - 79	21,061	6.80
		> 80	7,278	2.35
	Male	18 - 39	43,919	14.18
		40 - 59	46,892	15.14
		60 - 79	18,738	6.05
		> 80	4,429	1.43

Supplementary file

Shift in hospital opioid use during the COVID-19 pandemic in Brazil: a time-series analysis of one million prescriptions

S3 Table. Electronic-management systems of the 24 participating hospitals.

STATE	INSTITUTION	HIS[a]
Distrito Federal	Hospital Coração do Brasil	Tasy™
Distrito Federal	Hospital Santa Helena	Tasy™
Distrito Federal	Hospital Santa Luzia	Tasy™
Pernambuco	Hospital Esperança Olinda	WPD™
Pernambuco	Hospital Esperança Recife	WPD™
Pernambuco	Hospital Memorial São José	WPD™
Pernambuco	Hospital São Marcos	WPD™
Rio de Janeiro	Hospital Bangu	WPD™
Rio de Janeiro	Hospital Barra D'Or	WPD™
Rio de Janeiro	Hospital Caxias D'Or	WPD™
Rio de Janeiro	Hospital Copa D'Or	WPD™
Rio de Janeiro	Hospital Niterói D'Or	WPD™
Rio de Janeiro	Hospital Norte D'Or	WPD™
Rio de Janeiro	Hospital Oeste D'Or	WPD™
Rio de Janeiro	Hospital Quinta D'Or	WPD™
Rio de Janeiro	Hospital Rios D'Or	WPD™
São Paulo	Hospital São Luiz - Anália Franco	Tasy™
São Paulo	Hospital e Maternidade Assunção	Tasy™
São Paulo	Hospital e Maternidade Brasil	Tasy™
São Paulo	Hospital IFOR	Tasy™
São Paulo	Hospital São Luiz - Itaim	Tasy™
São Paulo	Hospital São Luiz - Morumbi	Tasy™
São Paulo	Hospital São Luiz - São Caetano	Tasy™
São Paulo	Hospital Villa Lobos	Tasy™

[a]HIS – Hospital Information System
Tasy™ - Philips Tasy EMR, Amsterdam, The Netherlands, (https://www.philips.ae/healthcare/resources/landing/tasy)
WPD™ - DGS Brasil - Dedalus Group, Barueri, Brazil (https://www.dedalusgroup.com/brasil/pt-pt)
